# Oleic Acid May Be the Key Contributor in the BAMLET-Induced Erythrocyte Hemolysis and Tumoricidal Action

**DOI:** 10.1371/journal.pone.0068390

**Published:** 2013-09-11

**Authors:** Mehboob Hoque, Sandeep Dave, Pawan Gupta, Mohammed Saleemuddin

**Affiliations:** 1 Interdisciplinary Biotechnology Unit, Aligarh Muslim University, Aligarh, India; 2 Institute of Microbial Technology (CSIR), Chandigarh, India; Instituto de Biociencias - Universidade de São Paulo, Brazil

## Abstract

A chance discovery of the tumoricidal action of a human milk fraction led to the characterization of the active component as oleic acid complex of the α-lactalbumin, which was given the acronym HAMLET. We report in this study that the oleic acid complex of bovine α-lactalbumin (BAMLET) is hemolytic to human erythrocytes as well as to those derived from some other mammals. Indirect immunofluorescence analysis suggested binding of BAMLET to erythrocytes prior to induction of hemolysis. Free OA was hemolytic albeit at higher concentrations, while sodium oleate caused hemolysis at far lower concentrations. Amiloride and BaCl_2_ offered protection against BAMLET-induced hemolysis suggesting the involvement of a cation leak channel in the process. BAMLET coupled to CNBr-activated Sepharose was not only hemolytic but also tumoricidal to Jurkat and MCF-7 cells in culture. The Sepharose-linked preparation was however not toxic to non-cancerous peritoneal macrophages and primary adipocytes. The tumoricidal action was studied using the MTT-assay while apoptosis induction measured by the annexin V-propidium iodide assay. Repeated incubation of the immobilized BAMLET with erythrocytes depleted oleic acid and decreased the hemolytic activity of the complex. Incubation of MCF-7 and Jurkat cells with OA, soluble or immobilized BAMLET resulted in increase in the uptake of Lyso Tracker Red and Nile red by the cells. The data presented support the contention that oleic acid plays the key role, both in BAMLET-induced hemolysis and tumoricidal action.

## Introduction

A serendipitous observation in the year 1995 that a casein fraction from human milk blocked the binding of bacteria to epithelial cell and was selectively toxic to a lung cancer cell line [1], evoked tremendous interest in the characterization of the tumoricidal molecule. The active fraction was soon traced to the casein fraction of human milk, obtained by acid precipitation [2] and was subsequently identified as oleic acid (OA)-bound partially unfolded α-lactalbumin (α-LA). The complex was given the acronym HAMLET (Human α-lactalbumin Made Lethal to Tumor cells) [3]. HAMLET exhibited broad tumoricidal activity against carcinomas, melanomas, glioblastomas and leukemias derived from human and various non-human sources [4]. Other studies showed that HAMLET like complexes with OA can also be prepared from bovine α-LA, which was designated as BAMLET [5], as well as from the protein derived from the milk of several other mammals [6]. The tumoricidal effect of HAMLET and BAMLET has been observed also in vivo, in animal models [4,7] and in a clinical investigation [8]. The exact mechanism by which HAMLET-like complexes exert tumoricidal action continues to remain elusive despite concerted global efforts. Available data however suggest that the OA complex of α-LA selectively enters tumor cells and induces apoptosis-like events, stimulates macroautophagy and inhibits proteasomes [9,10]. Storm et al. [11,12] using small-hairpin RNA inhibition, proteomic and metabolomics technologies showed that c-Myc oncogene is an important determinant of HAMLET sensitivity; cells expressing high levels of c-Myc were more sensitive, while knock down of the gene lowered HAMLET sensitivity. More recent studies showed that both prophylactic and therapeutic effects of HAMLET against colon cancer are mediated by β-catenin signalling [13].

Most studies show that native α-LA, used as control is not tumoricidal [4,5], but acquired the killer property only when converted to HAMLET. Several homologues of α-LA, fragments thereof [14] and even structurally unrelated proteins like β-lactoglobulin and parvalbumin also formed OA complexes that were selectively toxic to tumour cells [15]. A recombinant variant of human α-LA in which all cysteine residues were replaced with alanine and lacked tertiary structure was not tumoricidal on its own, but could be transformed into one after association with OA [16]. This suggested that both unfolding and OA binding are essential for the killer activity against tumor cells. Considering that wide variations in sequence and conformation of the α-LA do not negatively affect its transformation to an antitumor molecule and near specific requirement of OA, questions were raised on the seminal role of the protein in the HAMLET/BAMLET [14,15]. In a more recent study Brinkmann et al. [17] showed that some primary cells and even human erythrocytes are also sensitive to BAMLET and attributed the lytic activity primarily to the OA component. While other studies that support the contention that OA is the toxic principle and α-LA acts to facilitate its solubilisation appeared [18,19], these claims are contested by works which continue to attribute tumoricidal effect to the unfolded α-LA [11,12]. We have investigated in this study the effect of soluble and immobilized BAMLET on red blood cells as well as Jurkat and MCF-7 cell lines and observed that prevention of entry of α-LA component of the complex in the cells by coupling it to Sepharose support does not abolish hemolytic/tumoricidal action. The results support the contention that OA may be playing the seminal role in the toxic action of BAMLET on erythrocytes and at least some tumor cells.

## Materials and Methods

### Ethics statements

Blood was withdrawn by expert technician under the supervision of a senior physician in the University Medical College from volunteers. Goat and buffalo blood was collected from the local slaughterhouse, Frigerio Conserva Allana Ltd, Aligarh, from the routinely slaughtered animals after obtaining a written permission from the management. Blood from rats, mice and rabbits was collected from the institutional animal house (registration number 332) according to the recommendations of Committee for the Purpose of Control and Supervision of Experiments on Animals (CPCSEA), Government of India. The Aligarh Muslim University Bioethical Committee approved the use of blood from the slaughterhouse animals, animals maintained in the animal house and human blood from volunteers after obtaining their written consent on prescribed pro forma.

### Materials

Bovine α-LA (type III, Ca^2+^ depleted), Sodium Oleate (Na Oleate), Sepharose 4B and MTT assay reagent were obtained from Sigma Chemical Company, St. Louis, USA. OA was purchased from LOBA Chemie India. DMEM, RPMI, fetal bovine serum, penicillin–streptomycin and trypsin were purchased from Invitrogen. All chemicals not listed here were of analytical grade.

### Preparation of BAMLET

BAMLET was prepared by the heat-treatment procedure described by Kamijima et al. [20]. Briefly, α-LA was dissolved in phosphate-buffered saline (PBS), pH 7.4 to a concentration of 700 µM. OA (120 molar equivalents) was added to the protein solution, mixed gently and the mixture was incubated at 50 °C for 10 min. The tubes were cooled to 25 °C and centrifuged. After removal of excess OA by aspiration the preparation was subjected to dialysis against PBS for 18 h.

### Protein estimation

The concentration of α-LA was estimated spectrophotometrically. The molar extinction, *A* ^1%^_1 cm_ value of 20.1 (for native α-LA) and 22.8 (for α-LA in BAMLET) at 280 nm were used for calculating the concentration of protein [21].

### OA estimation

Concentration of OA was estimated by the procedure described by Wawrik and Harriman [22] with few modifications. Briefly, to Eppendorf tubes containing 400 µL of copper reagent (9 volumes (vol.) aq. 1.0 M triethanolamine, 1.0 vol. 1.0 N acetic acid, 10 vol. 6.45% (w/v) Cu(NO_3_)_2_.3H_2_O) was added equal vol. of OA containing samples prepared in 100 mM Tris-HCl buffer, pH 8.0 and the tubes were mixed by Vortexing. Chloroform (500 µL) was added and the tubes were Vortexed again for another 1 min. The samples were centrifuged at 14,000 x g for 3 min and the organic phase was carefully transferred into a new tube. Upon addition of 50 µL of 1% (w/v) sodium diethyldithiocarbamate in 2-butanol, yellow colour was developed which was read at 440 nm. For the preparation of standard curve OA was dissolved in ethanol and diluted in 100 mM Tris-HCl buffer, pH 8.

### Chemical cross-linking of α-LA and BAMLET

α-LA and BAMLET prepared as described earlier were chemically cross-linked following the method described by Spolaore et al. [19] with a few modifications. Briefly, 5 mg.mL^-1^ of protein in phosphate buffer (20 mM, pH 7.4) was incubated at 25 °C with 0.01% glutaraldehyde prepared freshly in the same buffer. The cross-linking reaction was stopped by adding 1 M Tris buffer, pH 7.4 at different time period and the samples were characterized by SDS-PAGE in presence of 1% β-mercaptoethanol. BAMLET was prepared from the cross-linked α-LA as described earlier.

### Immobilization of α-LA/ BAMLET on Sepharose 4B matrix

Sepharose 4B matrix was activated with cyanogen bromide (CNBr) and α-LA or BAMLET were coupled by the method of Porath et al. [23]. Appropriate quantities of α-LA or BAMLET were incubated with the CNBr activated Sepharose at 4 °C for 24 h. The matrices were washed thoroughly and the protein concentration measured in the wash supernatants. The amount of bound protein was calculated by subtracting the amount of protein in the wash from the initial amount added to the activated Sepharose matrix. The preparation thus obtained contained 4 mg protein per millilitre of the matrix. Immobilized α-LA was converted to BAMLET by the same procedure as described earlier for soluble preparation.

### Isolation of erythrocytes

Nine vol. of blood was collected from healthy volunteers in the tubes containing 2.7% (w/v) EDTA to prevent coagulation and centrifuged at 650 x g for 5 min. Plasma was removed carefully and the white buffy layer was completely removed by aspiration with a pipette with utmost care. The erythrocytes were then washed for additional three times in wash buffer (10 mM Tris, 0.9% NaCl and 5.0 mM glucose, pH 7.4) and used for further experiments.

### Effect of BAMLET on erythrocyte membrane

Erythrocyte membranes were prepared as described by Fairbanks et al. [24]. The cells were hemolyzed in 20 vol. of 5 mM Tris-HCl buffer, pH 8.0 at 4 °C. The membrane was then pelleted by centrifugation at 12,000 x g for 10 minutes at 4 °C and repeatedly washed with the buffer to prepare hemoglobin-free membranes. The membrane was treated with 70 µM BAMLET for 2 h. Membrane was also prepared from the BAMLET-treated erythrocytes and analysed by SDS-PAGE under reducing condition.

### Hemolysis assay

Ten microliters of packed erythrocytes were incubated with various concentrations of BAMLET for 90 min at 25 °C in a total vol. of 100 µL. The cells were then diluted to 1.0 mL with normal saline, incubated at 25 °C for 10 min. After removal of intact cells by centrifugation at 650 x g, haemoglobin in the supernatant was measured spectrophotometrically at 410 nm. For total lysis the erythrocytes were lysed in distilled water instead of saline and the value was used as 100 for calculation of percent hemolysis. All the experiments were reformed in quadruplets and repeated for at least three times.

### Indirect Immunofluorescence of BAMLET treated erythrocytes

Ten microliters of packed human erythrocytes were treated with 20 µM BAMLET for 10 min at 25 °C and washed with PBS, pH 7.4. The pelleted cells were incubated with polyclonal anti-BAMLET antibody raised in mice (1:100 dilution in PBS, pH 7.4) for 30 min at 37 °C. The cells were again washed with PBS, pH 7.4 and finally suspended in 100 µL of the buffer. FITC-conjugated rabbit anti-mouse IgG (Sigma) was added to the cell suspension at 1:100 dilution and incubated for 30 min at 37 °C in dark. The cells were washed thoroughly and visualized under fluorescence microscope using a 100X oil immersion objective (Zeiss M2 Imager; Zeiss, Göttingen, Germany).

### Cell culture

Cell lines were procured from cell repository at National Centre For Cell Science, Pune, India. Jurkat were grown in culture medium composed of RPMI-1640 and MCF-7 in DMEM with glutamine supplemented with 10 mM HEPES, 23.8 mM NaHCO_3_, 10% heat-treated fetal bovine serum, penicillin (60 µg.mL^-1^) and streptomycin (0.1 mg.mL^-1^). Cells growing in suspension (Jurkat) were kept at between 1 × 10^5^ to 1.5 × 10^6^ cells/mL. Cells were switched to serum-free medium 6 h prior to different treatment.

### MTT assay

MTT assay was performed as described previously [25]. Briefly, Cells were seeded at 5,000 cells/well on 96-well plates and incubated for 24 to 48 h before the treatment. Cells treatment was continued with or without stimuli for 24 h, a 20 µL aliquot of 3-(4,5-Dimethylthiazol-2-yl)-2,5-diphenyltetraolium bromide (MTT, a yellow tetrazole; 5 mg.mL^-1^ in PBS) was added to the wells and incubated for 4 h at 37 °C. After removing supernatant carefully, 200 µL of DMSO was added and mixed, and the absorbance was read at 563 nm.

### Staining with annexin-V and PI

Apoptosis studied by annexin V-FITC Apoptosis Detection Kit (Calbiochem) to stain cells in annexin binding buffer according to the manufacturer’s instructions. Cells were analyzed with a BD FACS Calibur flow cytometer (BD Biosciences).

### LysoTracker Red-uptake

Cells were cultured in media at 37°C and after treatment with or without compound. The acidotropic dye LysoTracker Red DND-99 was diluted in DMEM, resuspended in prewarmed (37 °C) medium containing 50 nM LysoTracker Red for 30 min [26]. Cells were then resuspended in PBS and lysosomal fluorescence of 5,000 cells per sample was determined by flow cytometry using the FL3 channel. CELLQUEST-PRO/Flowjo software was used to analyze all of the data from flow cytometric experiments.

### Nile Red Staining

Nile red staining was performed as described by Greenspan et al. [27]. Briefly, Nile red (1 mg.mL^-1^) in acetone was prepared and stored protected from light. The excitation wavelength was set at 488 nm with a 2 nm slit width; the emission wavelength was set at 540 nm with a 20 nm slit width and the fluorescence spectra were recorded. Excitation and emission fluorescence spectra were determined with a Cary Eclipse spectrofluorimeter (Varian). The relative fluorescence intensity of Nile Red in the presence of the various samples was obtained after subtraction of both the autofluorescence of the samples and the fluorescence intensity of Nile red alone in the buffer.

### Isolation of peritoneal macrophages and primary adipocytes

Murine peritoneal macrophages suitable for use in the experiments are isolated as described [28]. Seven/eight weeks old male inbred BALB/c pathogen-free mice were obtained from the animal house facility of the Institute of Microbial Technology (IMTECH), Chandigarh, India. Elicited peritoneal macrophages were harvested 4–5 days later of the mice immunization by intraperitoneal injection with 4% thioglycollate medium. Primary adipose cells were isolated from 8 to 9-week-old male C57BL/6J mice as described previously [29,30]. Briefly, the epididymal fat pads were removed, minced, and digested using collagenase at 37 °C for 2 h. The primary adipose cells were then washed extensively and incubated at 37 °C in Dulbecco’s modified Eagle’s medium containing 5% bovine calf serum.

### Statistical analysis

Results are expressed as the mean ± SD unless otherwise mentioned. SigmaPlot (SyStat Software) and SPSS (IBM) were used for statistical analysis. All statistical data were from averages of three or more independent experiments. Two-tailed Student t test was performed to obtain P values. Statistical significance was established at * P<0.01.

## Results and Discussion

**Figure 1:**
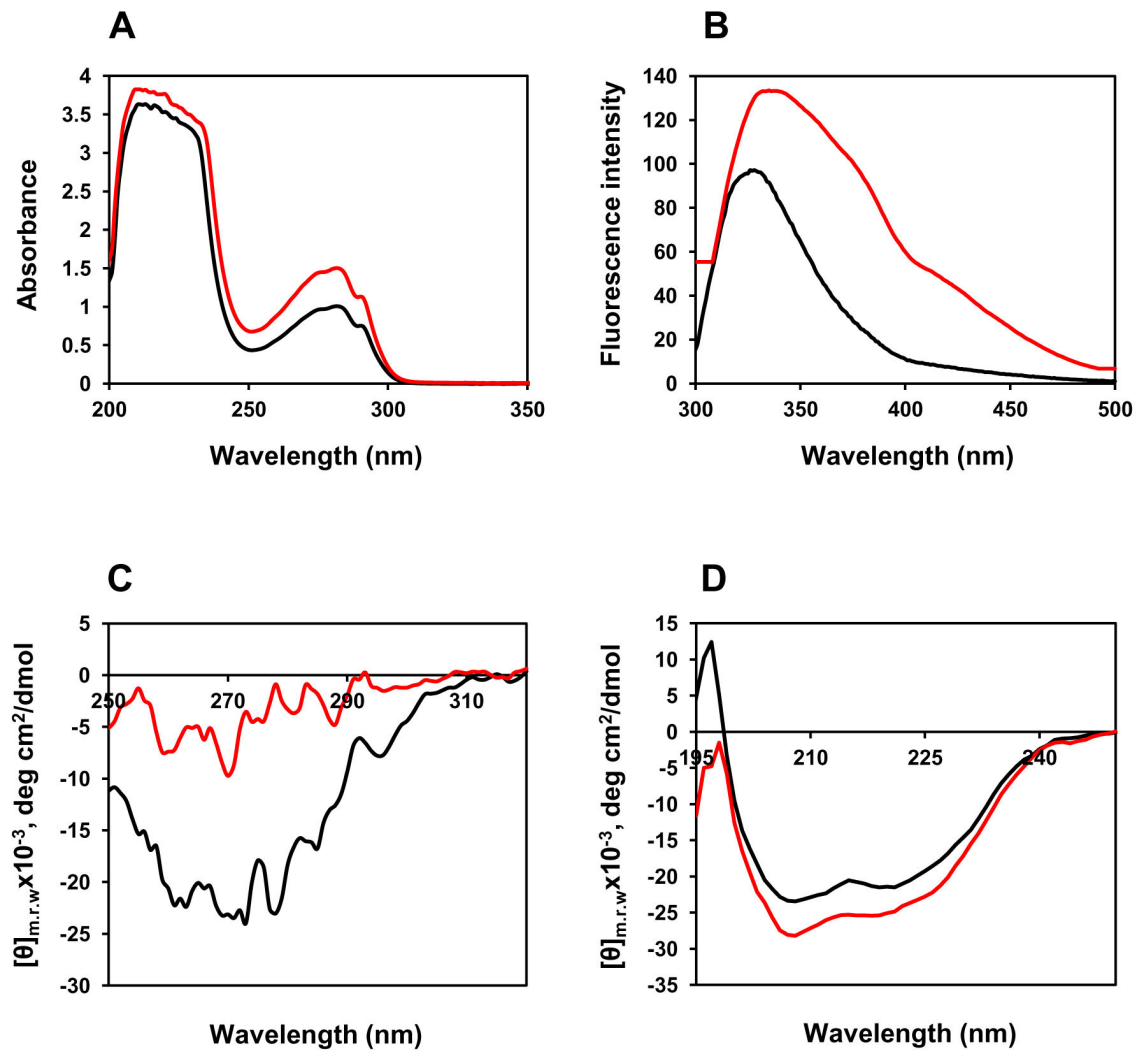
Spectrometric analysis of BAMLET (red line) native α-LA (black line). UV absorption spectra were recorded on a PerkinElmer Lambda 25 UV/VIS spectrophotometer in a quartz cuvette with 1.0 cm path length. Protein concentration used was 1.0 mg.mL ^-1^ (panel A). Intrinsic fluorescence spectra were recorded at 25 °C between 300 to 500 nm by exciting Trp at 295 nm in a Shimadzu RF-5301 PC spectrofluorophotometer using a quartz cuvette with 1.0 cm excitation path length. Protein concentration used was 0.1 mg.mL ^-1^ (Panel B). Near-UV CD spectra were recorded between 320 and 250 nm using quartz cuvette with 1.0 cm path length. Samples were prepared with a protein concentration 1.0 mg.mL ^-1^ (panel C). Far-UV CD spectra were recorded between 250 and 195 nm at the scan rate of 200 nm/min using a quartz cuvette with 0.1 cm path length. Protein concentration used was 0.3 mg.mL ^-1^ (panel D). The CD spectra were obtained using a JASCO J-815.

BAMLET used in the study was prepared following the procedure described by Kamijima et al. [20], in which heat-treated α-LA is exposed to OA. As also reported by the authors, BAMLET prepared thus exhibited altered spectroscopic properties, compared to α-LA, suggesting a molten globule like structure (Figure 1). While UV-absorption spectra indicate the exposure of side chains of aromatic residues to the solvent, loss of tertiary structure and acquisition of additional secondary structure are evident from the far and near UV-CD spectra. Intrinsic fluorescence measurements also suggest exposure of tryptophan(s) to the solvent and loss of tertiary structure. The structure of the complex bears striking similarity with that of BAMLET/HAMLET prepared by the ion exchange procedure [20]. The OA/protein molar ratio of the preparation was 10 (Table 1), which is close to that reported by Kamijima et al. [20], but an order of magnitude higher than the value reported by Svensson et al. [5] for the complex originally prepared by the ion-exchange procedure. More recent studies however suggest that in HAMLET-like complexes, obtained using various procedures including that based on ion-exchange, each molecule of α-LA binds to multiple molecules of OA [31]. In a very recent study Nakamura et al. [32] compared stoichiometry of protein and OA in HAMLET prepared in the authors’s laboratory by the ion-exchange procedure [3] and the heat-treatment procedure [20], also used in this study. Both the preparations contained 8.5 molecules of OA per protein molecule, a value close to that observed by us in this study.

**Table 1:** α-LA and OA molar ratios in various BAMLET preparations.

**BAMLET Preparation**	α-LA:OA molar ratio*
Uncrosslinked	1: 10 (±1.4)
Crosslinked prior to OA binding	1: 11 (±1.52)
Crosslinked after OA binding	1: 10 (±1.4)

* Each value represents the mean ±SD of four determinations

**Figure 2:**
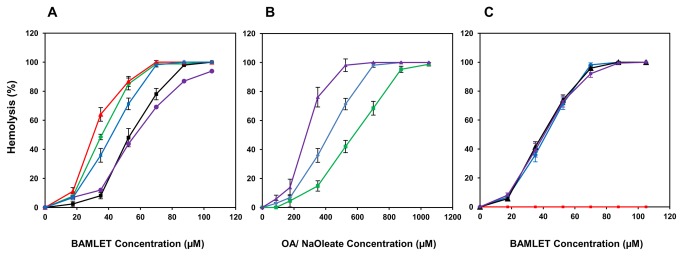
BAMLET induced hemolysis of erythrocytes. Erythrocytes isolated from the blood of mouse (purple line), goat (black line), human (blue line), rabbit (green line) and buffalo (red line) were incubated with BAMLET and the resulting hemolysis determined as described under methods (Panel A). Human erythrocytes were treated either with free OA (green line), BAMLET (blue line), and free Na oleate (purple line) and the resulting hemolysis determined (panel B). Human erythrocytes were incubated with BAMLET (blue line), glutaraldehyde-treated BAMLET (purple line), and glutaraldehyde-treated α-LA converted to BAMLET (black line) and crosslinked α-LA (red line) (Panel C). Data are expressed as mean ± SD.

**Figure 3:**
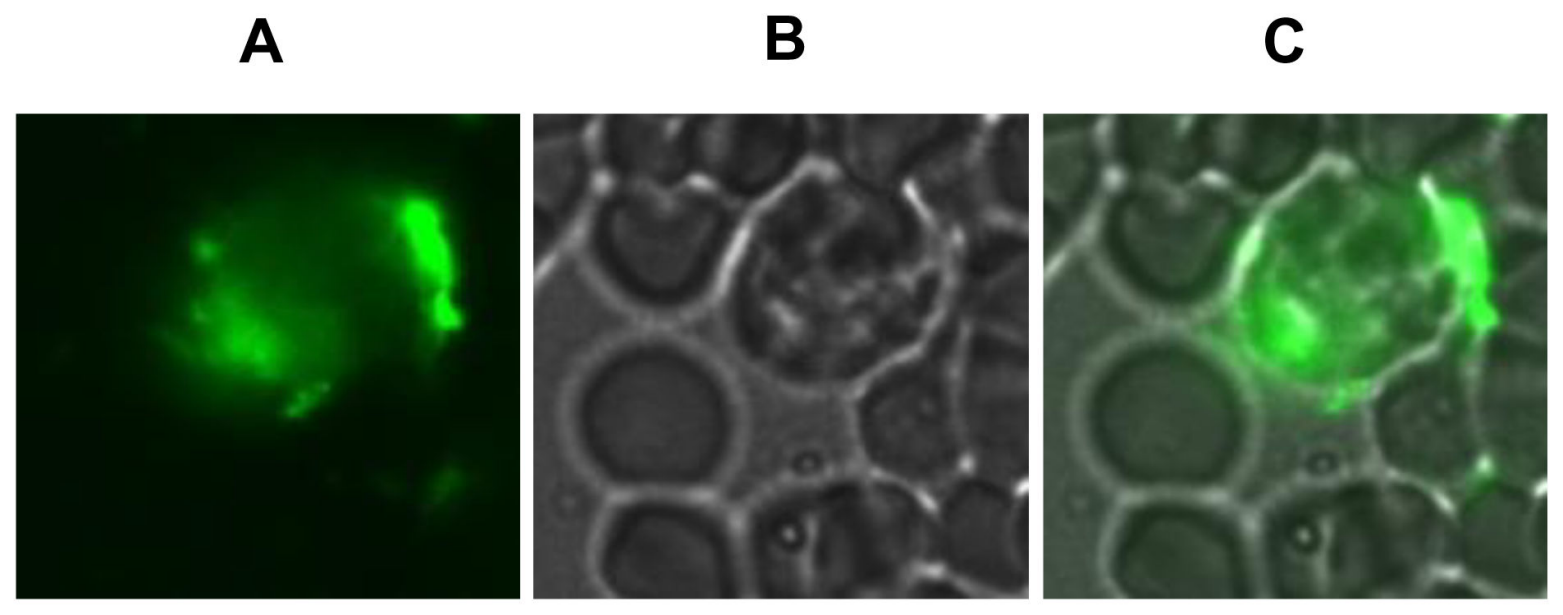
Interaction of BAMLET with erythrocytes. Human erythrocytes were incubated with 20 µM BAMLET in PBS for 10 min at 25 °C and washed thrice with PBS, pH 7.4. Anti-BAMLET polyclonal antibodies raised in mouse were added and the cells were further incubated at 37 °C and again washed thoroughly. The interaction was then probed with FITC conjugated secondary rabbit anti-mouse IgG (Sigma Chemical Co.) and the cells visualized under a fluorescence microscope at 100X magnification. The images shown are those observed under fluorescence (Panel A), bright field (Panel B) as well as that merged of both fluorescence and bright filed (Panel C).

While studies showing selective killing of cancer cells by BAMLET are numerous [1,4,33,34], recent reports suggest that the OA complex of α-LA may also be toxic to some non-cancerous cell, human erythrocytes [17] as well as to antibiotic sensitive and resistant strains of *Streptococcus* *pnuemoniae* [35,36]. Figure 2A shows that in addition to human erythrocytes, those derived from several other mammals were readily hemolyzed by BAMLET, although their sensitivity to the α-LA-OA complex varied. While buffalo erythrocytes were most susceptible, those from mouse exhibited maximum recalcitrance, requiring 100 µM BAMLET for causing complete hemolysis. Ruminant erythrocytes lack phosphatidyl choline, the prominent phospholipid of most mammalian red cells [37], yet of the two ruminant erythrocytes investigated buffalo erythrocytes were highly susceptible and goat erythrocytes more resistant to BAMLET-induced hemolysis. This suggests that hemolytic action of BAMLET may not necessitate specific composition of the membrane lipid(s). Toxic action of BAMLET against some bacteria [35,36] with membranes differing remarkably in lipid composition also argues against specific role of membrane lipids. Some studies have however provided evidence that nature of membrane lipids may determine the mode of action of HAMLET/BAMLET. HAMLET binds uniformly to the vesicles prepared from egg yolk and soybean phospholipids but when exposed to those prepared from tumor cell membranes reveal punctate binding pattern [38] and influence the permeability of tumor cell membranes to ions [1,39]. In a recent study it was shown that *S. pneumoniae* D39 cell membrane damage caused by BAMLET was mediated through Na^+^/Ca^+2^ exchange activity, calcium entry and membrane depolarization [15]. The indirect immunofluorescent images of BAMLET treated erythrocytes provided evidence for the binding of BAMLET to erythrocyte surface (Figure 3). The BAMLET binding was detectable after incubation of the human erythrocytes with mouse anti-BAMLET antibody followed by FITC conjugated secondary antibody. The fluorescence was however lost on longer incubation due to hemolysis of the cells. We have however noticed no significant binding of BAMLET to human erythrocyte membrane proteins by SDS-PAGE after incubation of the intact cells or hemoglobin-free ghosts with the complex (Figure S1).

A recent report suggests that HAMLET forms annular oligomers when deposited on phospholipid monolayers, a property shared by α-LA and the peptide C derived from the protein but only at higher concentrations [40]. While studies that provide evidence supporting the formation of annular oligomers in intact cells exposed to HAMLET/BAMLET are lacking, the authors envisage a role of the annular oligomers in the permeability alterations and accumulation of the complex observed cytoplasm and subcellular organelles of the HAMLET exposed tumor cells.

Free OA was hemolytic but caused the lysis of human erythrocytes at concentrations higher than those required when present in the form of complex with α-LA (Figure 2B). Brinkmann et al. [17], however observed OA to be equally hemolytic in free form or when associated with the protein. While we have no definite explanation for the discrepancy, poor solubility of OA, its tendency to stick to the walls of plastic tubes under the conditions of the study and consequent low availability [17] for interacting with the cells apparently necessitated high concentrations of the fatty acid to induce hemolysis. Oleate, that forms HAMLET-like complexes [41] and its relevant constituent [42], was however more hemolytic than OA and moderately more hemolytic than even α-LA associated OA (Figure 2B). BAMLET prepared by the procedure of Kamijima et al. [20], when subjected to crosslinking with 0.01% glutaraldehyde resulted in the formation of adducts that moved in SDS-PAGE as polypeptides ranging from 50 kDa to those that were too large to enter the gel (Figure S2) as also reported by Spolaore et al. [19]. The amount of OA associated with the crosslinked preparations containing mostly protein oligomers was comparable with that of the uncrosslinked BAMLET (Table 1). Both crosslinked BAMLET or α-LA that was heat-treated and crosslinked with glutaraldehyde prior to binding of OA were also hemolytic (Figure 2C) suggesting a mechanism of hemolytic action that may not require the entry of the BAMLET as entry of the covalently linked protein aggregate in the erythrocytes is unlikely, due to the inability of the erythrocytes to take up large molecules by phagocytosis/ endocytosis.

**Figure 4:**
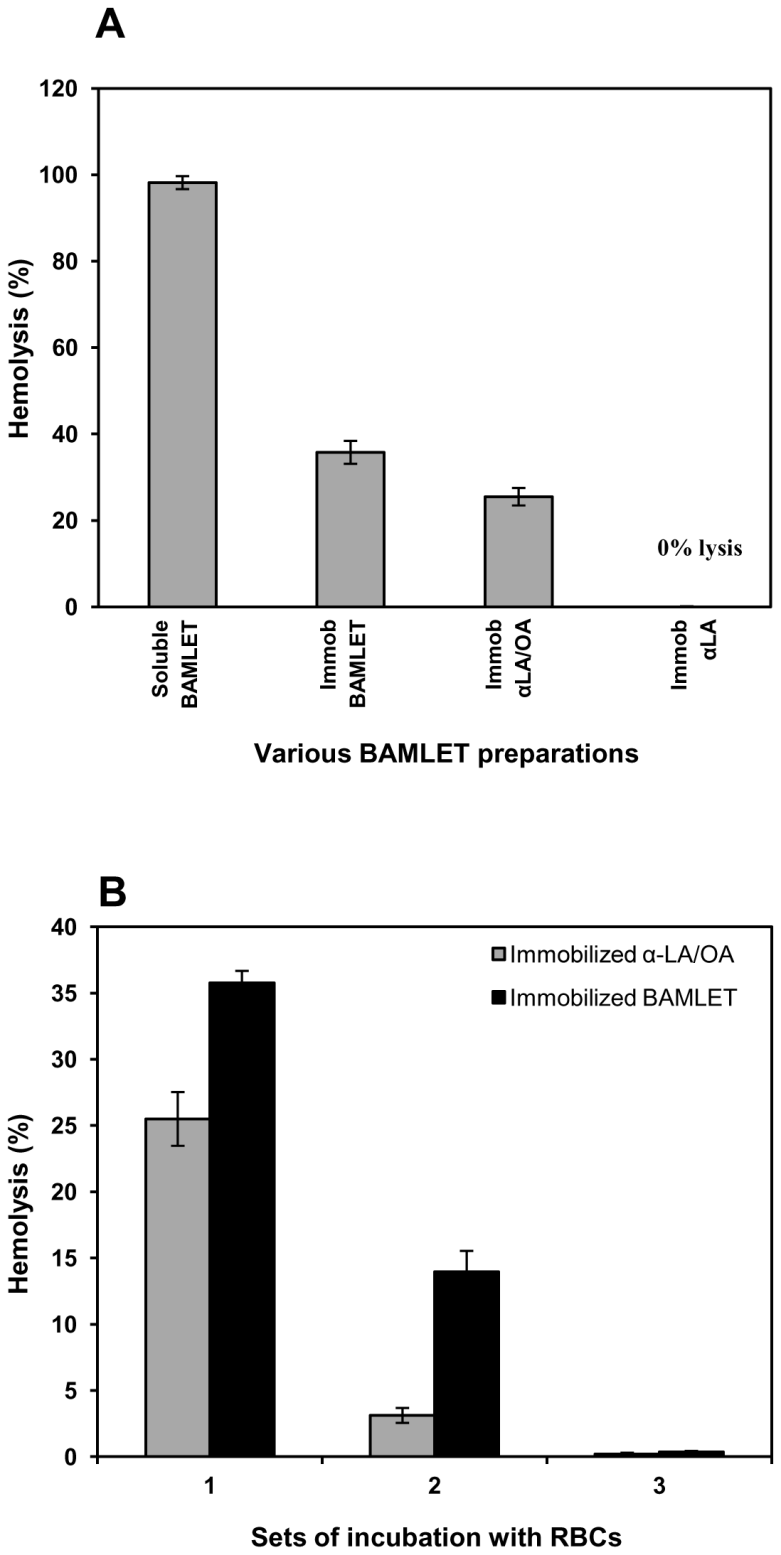
Hemolysis of human erythrocytes incubated with soluble and immobilized BAMLET. Human erythrocytes were incubated with soluble BAMLET, immobilized α-LA, immobilized BAMLET and immobilized α-LA converted to BAMLET and the resulting hemolysis quantitated (panel A). Erythrocytes were incubated with immobilized BAMLET or immobilized α-LA converted to BAMLET and hemolysis quantitated. The immobilized preparations were collected, washed and incubated with a fresh batch of erythrocytes. The immobilized preparations were reused after the third cycle (panel B). Data are expressed as mean ± SD.

In order to further investigate the relative roles of α-LA and OA in inducing the hemolysis, BAMLET was immobilized on Sepharose 4B and hemolytic property of the immobilized preparation on human erythrocytes investigated. The idea was to prevent the entry of the protein component of the complex into the erythrocytes and restrict its action to the cell surface. OA binding was not affected when BAMLET was coupled onto Sepharose and the matrix bound α-LA was equally effective in binding the fatty acid when carried through the procedure of BAMLET formation (Table 2). As shown in Figure 4A, both the immobilized preparations were hemolytic towards human erythrocytes, albeit to a lower extent than the soluble BAMLET. While conformational alterations in α-LA resulting from the immobilization were not studied, the ability of the immobilized α-LA to bind OA and the resulting complex to hemolyze erythrocytes is indicative of conformational similarity between the BAMLET and the Sepharose-immobilized preparation. Since immobilized BAMLET preparations did not leach out any significant amount of the protein, when incubated in absence of erythrocytes for up to 12 h, as analysed by ELISA (detection limit 0.16 ng), using specific anti-α-LA polyclonal antibodies that also recognize BAMLET (data not included), it is unlikely that protein released from the Sepharose support contributed towards hemolysis.

**Table 2:** α-LA/OA molar ratios in the immobilized α-LA complexes of OA after incubation with human erythrocytes^*^.

**BAMLET Preparation**	**No incubation**	**Single incubation**	**Two incubations**	**Three incubations**
Sepharose-linked α-LA incubated with OA	1:10 (±1.6)	1: 5 (±0.81)	1: 2 (±0.82)	1:0
Sepharose-linked	1:10 (±1.53)	1: 6 (±0.78)	1: 3.5 (±0.58)	1:0

* Each value represents the mean ±SD of four determinations

Studies on the reuse of the immobilized BAMLET further supported the seminal role of OA (Figure 4B). In these studies, the immobilized BAMLET preparations, after incubation with erythrocytes, were collected, washed and reincubated with a fresh batch of erythrocytes. The hemolytic activity decreased remarkably after second incubation and disappeared completely after the third. Analysis of bound OA suggests that the OA content of the immobilized BAMLET preparations decreased remarkably after first exposure to erythrocytes and became almost undetectable after the second (Table 2).

**Figure 5:**
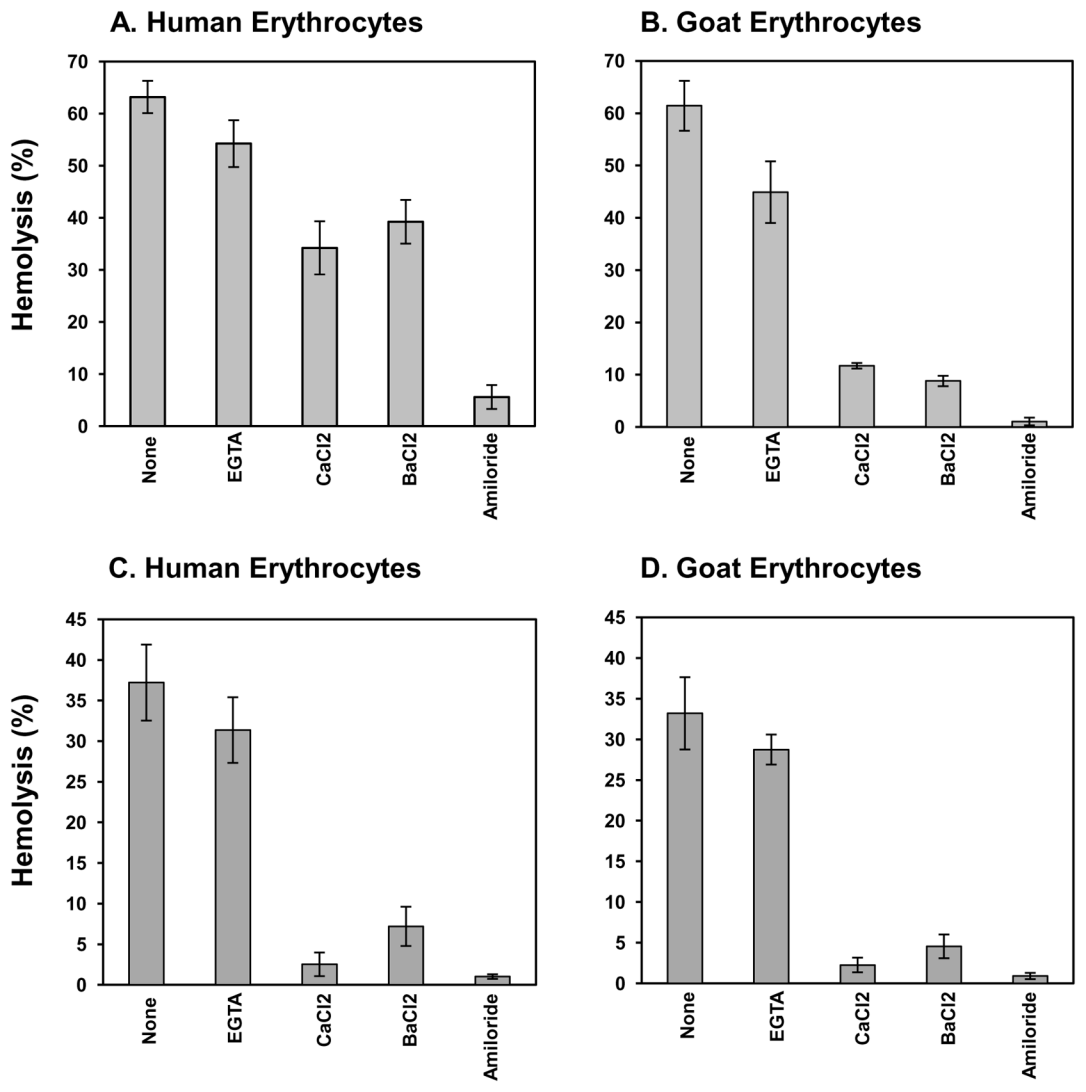
Effect of calcium, EGTA and ion-channel inhibitors on BAMLET-induced hemolysis of erythrocytes. Human (Panel A & C) and goat (Panel B & D) erythrocytes were incubated with 1 mM of each the test substances for 30 min at 25 °C and 50 µM soluble (Panel A & B) or 70 µM immobilized BAMLET (Panel C & D) were added. The samples were further incubated for additional 90 min at 25 °C and the resulting hemolysis measured. The results shown are average of three independent experiments, which were each conducted in triplicate. Data are expressed as mean ± SD.

Storm [12] observed that HAMLET induces activation of an ion channel involved in the transport of K^+^, Na^+^ and Ca^2+^ in the membranes of tumor cells that explains the earlier observed intracellular calcium accumulation by the tumor cells exposed to the complex. Interestingly, neither OA nor native α-LA caused the ion channel activation. More recent studies from the group suggest that enhanced ion fluxes across the plasma membrane are essential for the HAMLET-induced initiation of the processes that eventually lead to tumor cell death [43]. Studies employing lymphomas and carcinomas also suggest the involvement of a K^+^, Na^+^ and Ca^2+^ permeable non-selective cation channel, distinct from the classical TRP, ENaC- or CNG channels in HAMLET-mediated cell death. Fully differentiated HRTEC cells however do not reveal comparable ion channel activation in response to HAMLET. Our studies also suggest the possible involvement of a non-specific cation channel activation in the hemolysis induced both by soluble (Figure 5A & B) and Sepharose bound BAMLET (Figure 5C & D) in human as well as goat erythrocytes. The BAMLET-induced hemolysis was preceded by cation leakage and inhibited by BaCl_2_ and amiloride, suggesting similarity with several non-specific ion channels and inhibitors. Also while inclusion of EGTA moderately decreased the susceptibility of erythrocytes to the BAMLET-induced lysis, indicating a role of Ca^2+^, inclusion of CaCl_2_ in the medium was protective against hemolysis. Ca^2+^ release OA from BAMLET [19], form insoluble salt and lower the availability of OA for interaction with the membrane [44]. The sensitivity of soluble and Sepharose-bound BAMLET-induced hemolysis to the inhibitors suggests similarities in their mechanism of action. Similarly OA-induced hemolysis was also sensitive to BaCl_2_ (data not included). Evidence for the existence of non-selective cation channels, by applying patch-clamp single chain recording have been provided in human erythrocytes [45] and these were further characterized with respect to the stimulants [46,47]. More recently Föller et al. [48] have shown that human and mouse erythrocytes contain a transient receptor potential channel that contributes towards cation leak. During their investigation of HAMLET-induced ion channels in A549 lung carcinoma cells, Storm et al. [43] observed that the increase in free intracellular calcium was not restricted by inclusion of EGTA in the medium but by the inhibition of INsP3-gated ER Ca^2+^ channel with U73122, implying that it originated mainly from intracellular stores. In another recent study [42] it was however shown that HAMLET and oleate trigger overlapping but fundamentally different responses on the ion channels of the lung carcinoma and Jurkat cells.

**Figure 6:**
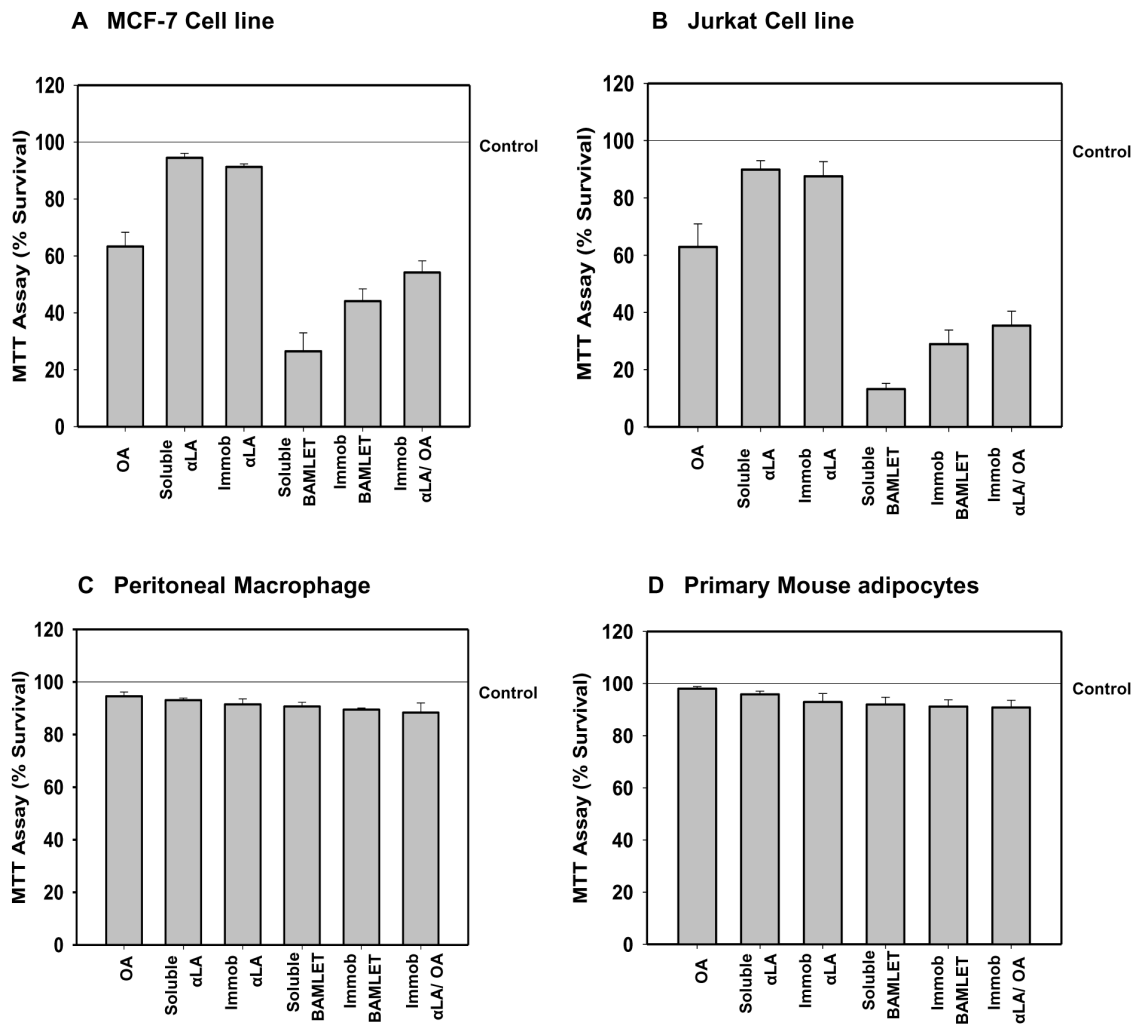
Cytotoxicity of immobilized BAMLET. The cytotoxicity of free OA, soluble αLA, immobilized αLA, soluble BAMLET, immobilized BAMLET and immobilized αLA converted to BAMLET was evaluated on tumor cell line (A) MCF-7 and (B) Jurkat cells and primary cells (C) Mouse Peritoneal Macrophage and (D) Primary Mouse adipocytes, with the MTT assay after incubation for 24 h with mild shaking. The results were confirmed by three independent experiments, which were each conducted in triplicate. Data are expressed as the mean ± SD.

**Figure 7:**
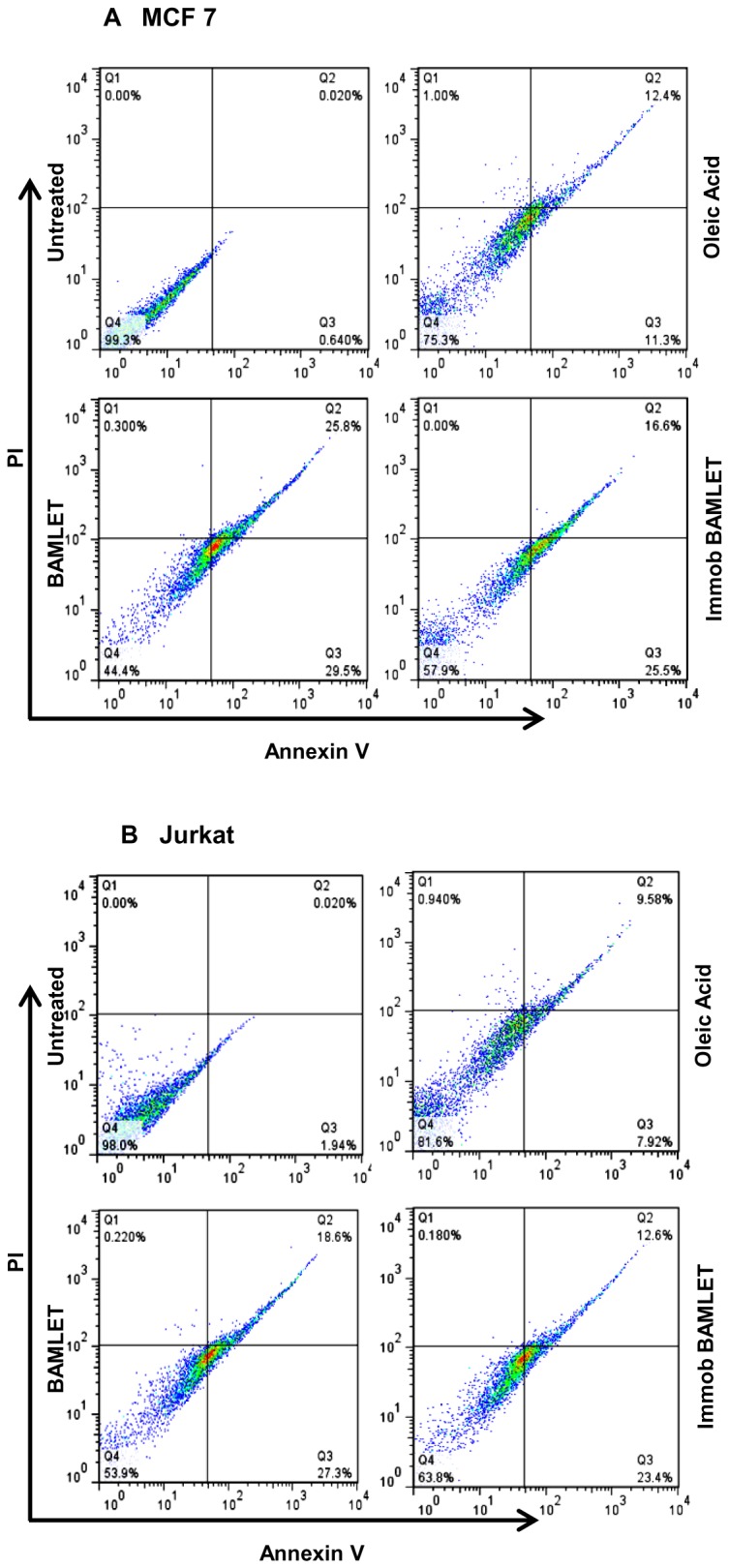
A comparison of BAMLET-induced apoptosis and necrosis in MCF-7 (A) and Jurkat cells (B). Cells were incubated with immobilized-BAMLET, soluble BAMLET or OA for 24 h with mild shaking before flow cytometric analysis. OA was dissolved in ethanol, diluted in media and was then sonicated before treatment to the cells. The results were confirmed by three independent experiments, which were each conducted in triplicate. Data are expressed as the mean ± SD. *P<0.01 vs. controls.

Immobilized BAMLET was also tumoricidal against MCF-7 and Jurkat cells. The MTT assay indicates that, unlike the soluble and immobilized α-LA which were barely toxic, BAMLET markedly lowered the number of surviving cells when added either as soluble or Sepharose bound preparations (Figure 6A & B). Neither decrease in cell viability nor apoptosis induction was however evident when non-cancerous mouse primary adipocytes or peritoneal macrophages were incubated with the soluble or immobilized BAMLET preparations (Figure 6C & D). The annexin-V staining assay, that detects apoptotic cell memebrane phosphatidyl serine (PS) externalization [49], was employed to further examine if the observed decrease in cell viability is related to induction of apoptosis-like processes [50]. Figure 7 reveals that the decrease in the viability of Jurkat and MCF-7 cells is indeed related to induction of apoptosis-like process by BAMLET with large fraction of cells exposed either to soluble or immobilized complex exhibiting enhanced PS exposure.

**Figure 8:**
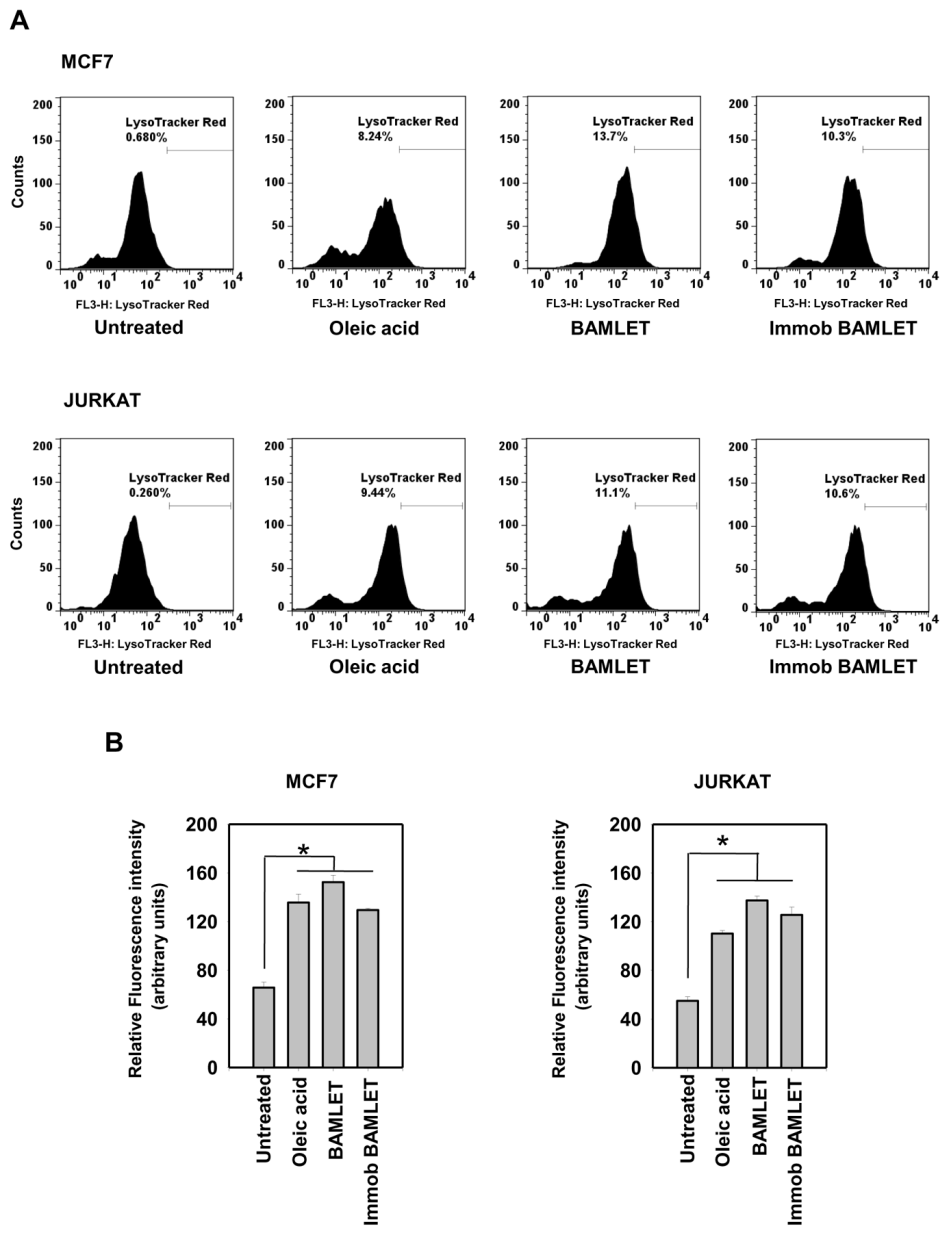
Effect of soluble and immobilized BAMLET on the uptake of Lyso Tracker and Nile Red. (A) LysoTracker signal in the MCF-7 (Top panel) and Jurkat (Bottom panel) cells after 4 h of incubation with OA, BAMLET or immobilized BAMLET. Cells were labelled with LysoTracker and the signal was analyzed by flow cytometry as described in Methods. (B) Nile red uptake by MCF-7 (left panel) and Jurkat (Right panel) cells after 4 h incubation with OA, BAMLET, and immobilized BAMLET. The results were confirmed by three independent experiments, which were each conducted in triplicate. Data are expressed as the mean ± SD. *P<0.05 vs. Controls.

BAMLET has been shown to induce apoptosis in the MCF7 and Jurkat cell lines [17,51] the cell death is caspase-independent being insensitive to caspase inhibitors and proceeding unhindered in caspase 3-deficient cells [51,52]. HAMLET-like complexes have been shown to have multiple targets including the plasma membrane, mitochondria, lysosomes, proteasomes, endoplasmic reticulum, histones in nuclei [52,53] and some metabolic pathways [11] in tumor cells. Some reports suggest that cytotoxicity of the HAMLET-like complexes may not involve either a classical apoptosis mechanism or autophagy but lysosomal membrane permeabilization and resulting leakage of the digestive enzymes cathepsin [51]. Figure 8A shows the accumulation of Lysotracker Red in the lysosomes of both MCF-7 and Jurkat cells four hours after treatment with soluble and immobilized BAMLET as well as free OA, suggestive either of lysosomal acidification. Longer incubation however made Lysotracker staining complicated particularly in MCF7 cells perhaps because of permeabilization of lysosome as also shown earlier [51]. This suggests that lysosomal acidification precedes permeabilization of lysosomes especially in MCF7 cells. Lysosomal acidification has earlier been correlated to leaky monovalent permeable ion channel TRP-ML1 which is involved in control of pH in the organalle [54]. Also Cadmium induced tandem acidification and permeabilization of lysososmes has been reported [55]. It is tempting to suggest of a possible role of the cation channel in acidification and permeabilization of lysosomes in the BAMLET/OA-treated cells. Soluble and immobilized BAMLET-mediated increase in Nile Red uptake (Figure 8B) is suggestive of oleic acid access to the cells. HAMLET induced uptake of OA by cancer cells has also been demonstrated in an earlier study as well [11]. A corollary question is whether BAMLET has cell specific attribute and mechanism to induce cell death remain unanswered. There has been a spectrum of multipronged reports implicating the cytotoxicity of the BAMLET to tumor cells by different mechanisms including apoptosis, necrosis, autophagy, perturbance in the chromatin structure, proteosome degradation, mitochondrial membrane depolarization, cytochrome C release, PS exposure, and a low caspase response [52]. In its simplest rendition, this complexity might underlie as few of these mechanisms associated with specific cell lines, although different mechanisms operative in a single cell lineage to variable parameters such as concentrations and different time points have also been suggested.

The ability of OA to exert tumoricidal activity on its own has been documented in some early and more recent studies [56-59]. Antitumor potential of OA was recognized several decades earlier [60,61] and its protective role against various forms of cancer has been supported by epidemiological studies [62,63]. The central role of OA in killing of the tumor cells was also envisaged from the observations that α-LA isolated from milk of several mammals and differing to varying extent in amino acid sequence [6], mutants of the protein [16] and even those structural unrelated [15], could be converted to tumoricidal complexes, while OA requirement was almost exclusive. More direct evidence in favour of the seminal role of OA in the toxic action of HAMLET/BAMLET was provided by Permyakov et al. [15], on the basis of their studies on OA complexes of few proteins structurally and functionally different from α-LA, both on Hep-2 tumor cell cells and *S. pneumoniae*. Also considering the observed decrease in thermal stabilization that accompanied complex formation of β-lactoglobulin with OA after exposure to high temperature, even the requirement for molten globule like conformation of the protein in the complexes to exert killer action against tumor cells was questioned [15].

Menendez et al. [56] observed that OA suppresses the Her-2/neu overexpression and in turn interacts synergistically with anti-Her-2/neu breast cancer immunotherapy by promoting apoptotic death of breast cancer cells exhibiting amplification of the Her-2/neu gene. Khamaisie et al. [59] reported that OA inhibited Bcr-Abl kinase autophosphorylation in Ba/F3 cells and showed anti-CML activity in a BCR/ABL-positive mouse model. Interestingly the Gas Chromatography-Mass Spectrometry (GC-MS) profiles of cellular metabolites of A549 lung carcinoma cells incubated with HAMLET revealed rapid uptake of OA from the complex [11].

It is now recognized that unfolded α-LA facilitates the solubilisation/dissociation of large aggregates of ionized fatty acid that are formed above the critical micellar concentration [14,17,19]. The complex prepared using different procedures comprise between 1 to 20 molecules of OA per α-LA depending on the procedure used and the fatty acid may bind at multiple sites [64]. Binding to the protein is likely to facilitate availability of higher and toxic concentration of OA for erythrocytes and tumor cells. The studies of Permyakov et al. [15] have also showed a direct correlation between the fatty acid concentration in protein complexes and toxicity.

A large number of studies have however shown that the partially unfolded α-LA present in HAMLET/BAMLET plays important role in its toxicity against the tumor cells. Evidence for the penetration of HAMLET/BAMLET into tumor cells and attack on several critical organalles including mitochondria, endoplasmic reticulum, proteasomes, lysosomes and trigger cell death pathways are available [9,34,53]. Storm et al. [11] observed that that c-Myc oncogene is an important determinant of HAMLET sensitivity; cells expressing high levels of the c-Myc gene were more sensitive while knock down of the gene lowered HAMLET sensitivity and modified the glycolytic state of the cells. The *in vivo* interaction of Alexa Fluor 568-labelled HAMLET with hexokinase1 using confocal microscopy as well as direct binding to the enzyme *in vitro* with the help of a high functional protein array has also been taken as evidence in support of the tumoricidal role of α-LA in the HAMLET [11]. A recent review has also summarized the potential role of the partially unfolded protein in HAMLET/BAMLET-like complexes [34]. Puthia et al. [13], in a more recent study have shown that HAMLET acts by triggering a Wnt-independent and ion channel dependent mechanism controlling β-catenin level in addition to the effects on β-catenin signalling.

Nakamura et al. [32] in recent study characterized that OA binding site in HAMLET and a similar complex prepared using goat α-LA (GAMLET) by 2D NMR spectroscopy and observed remarkable differences in OA binding sites; in HAMLET most of the residues affected by OA binding are within the A- and B- helices while those of GAMLET are located between the α and β subdomains. This suggested little role of α-LA, as no unique OA binding structure may be essential in the protein for expression of antitumor activity. In contrast, another very recent report concluded, based on a comparative analysis of ion fluxes, gene expression and tumoricidal activity that oleate and HAMLET produce overlapping yet clearly distinct effects [42].

Most studies describing tumoricidal action of HAMLET observed little or no toxicity of native α-LA against the tumor cells. Our studies on human erythrocytes and MCF-7 and Jurkat cells substantiate these observations (Figure 6A and B). Some earlier studies however show that native α-LA may induce apoptosis at least in some cells. For example native α-LA has been shown to cause apoptosis of mouse, cape fur seal and human mammary and cell lines as well as fibroblasts and myoepithelial cells and the activity was lost on heat treatment and pepsinization [65]. Apoptosis induction by native α-LA in human colon adenocarcinoma [66] and RAW 264.7 cells [67] has also been documented.

Erythrocyte hemolysis and tumoricidal action of HAMLET/BAMLET may involve different mechanisms of action. Unlike in case of the tumor cells, erythrocyte hemolysis as shown by experiments with immobilized preparations, may be mediated through the action of BAMLET or the bound OA principally on the cell surface molecules. Cytosolic events requiring genomic involvements are essential for causing apoptosis-like cell death in case of the tumor cells. Although BAMLET binds to erythrocytes (Figure 3), its possible entry in to the cells could not be studied by ELISA of the protein due to ensuring hemolysis. HAMLET has been shown to lose its tumoricidal activity in presence of serum albumin [68]. Characterization of an equine lysozyme multimeric complex with OA, a preparation resembling HAMLET but with higher OA: protein ratio, subsequent to interaction with membranes reveals conversion to nearly native like state, apparently after offloading of the bound fatty acid [69]. It is therefore likely that HAMLET/BAMLET may be stripped of the bound OA during its interaction with the target cells. Unfortunately no study describing the nature of α-LA after internalization of the complex is available.

In a recent study Permyakov et al. [15], assuming the low contribution of the protein component in HAMLET towards its tumoricidal action and ability of proteins widely differing in structure to form BAMLET-like complexes suggested that association of OA with established antitumor proteins may generate superior tumoricidal molecules. Native α-LA can also be tumoricidal at least against some cells as discussed earlier and possible presence of even OA-depleted BAMLET in the cells may also enhance the killer action of OA. The observed moderately low effect of immobilized BAMLET, as compared to the soluble complex (Figure 6A & B) may indicate such supplementation of the OA-induced tumoricidal action by the protein. To the best of our knowledge the exact nature of structure assumed by α-LA after the entry of BAMLET/HAMLET in the tumor cells is not known. It is however not likely that it will remain in the form of complex, because interaction with the membrane and cytoplasmic proteins that may deplete the molecule of its OA. Characterization of the structure of BAMLET internalized by the tumor cells will certainly be interesting.

In conclusion, this study provides evidence that OA component of BAMLET may play a key role both in causing hemolysis of mammalian erythrocytes as well as in the killing of Jurkat and MCF-7 cells. This was apparent from the experiments which showed that coupling of BAMLET to Sepharose support did not abolish the hemolytic and tumoricidal activities. Immobilized BAMLET exposed to erythrocytes was depleted both of bound OA and the ability to hemolyze erythrocytes. A recent exhaustive review by Fontana et al. [31] provides several arguments to show that OA can exert nearly all cytotoxic effects reported for HAMLET-like complexes and tends to conclude that the protein moiety does not possess toxic effect on its own. It is however likely that OA additionally facilitates the entry of α-LA in to the cells which after attaining a specific conformation may act to enhance the tumoricidal action.

## Supporting Information

Figure S1 **SDS-PAGE of the membranes prepared from erythrocytes incubated with BAMLET.** Membranes were prepared from control and BAMLET-treated human erythrocytes. The lane M contained molecular weight markers, Lane 1, membrane prepared from control erythrocytes; Lane 2 and 3, membranes prepared from hemolyzed and unhemolyzed erythrocytes after incubation with BAMLET respectively. Lane 4 contained human erythrocyte membranes incubated with BAMLET. 20 µg of membrane protein was applied in each lane.
(TIF)

Figure S2 **SDS-PAGE of glutaraldehyde treated α-LA (A) and BAMLET (B).** α-LA and BAMLET were subjected to glutaraldehyde treatment as described under methods. Lane M: protein molecular weight marker; Lane 1: uncrosslinked α-LA/BAMLET; Lane 2, 3, 4, 5 and 6 contain the preparations crosslinked for 1, 5, 30, 60 and 360 min, respectively.
(TIF)
