# Supplementary figures and images for: Oleic Acid May Be the Key Contributor in the BAMLET-Induced Erythrocyte Hemolysis and Tumoricidal Action
Source: PLoS One. 2013 Sep 11;8(9):e68390. doi: 10.1371/journal.pone.0068390 (PMC3770648; doi:10.1371/journal.pone.0068390)

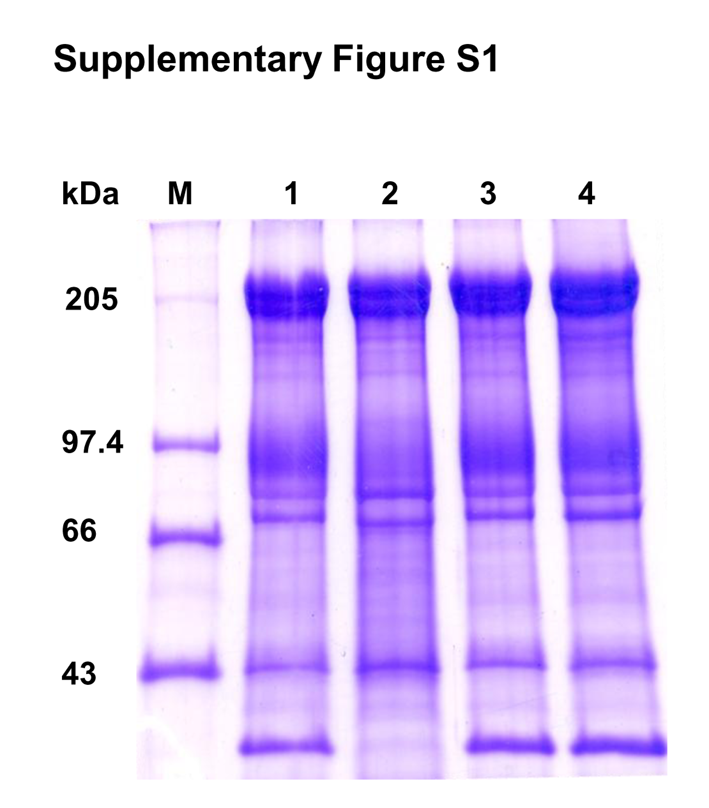

Supplement: Figure S1 — SDS-PAGE of the membranes prepared from erythrocytes incubated with BAMLET. Membranes were prepared from control and BAMLET-treated human erythrocytes. The lane M contained molecular weight markers, Lane 1, membrane prepared from control erythrocytes; Lane 2 and 3, membranes prepared from hemolyzed and unhemolyzed erythrocytes after incubation with BAMLET respectively. Lane 4 contained human erythrocyte membranes incubated with BAMLET. 20 µg of membrane protein was applied in each lane. (TIF) [file pone.0068390.s001.tif]

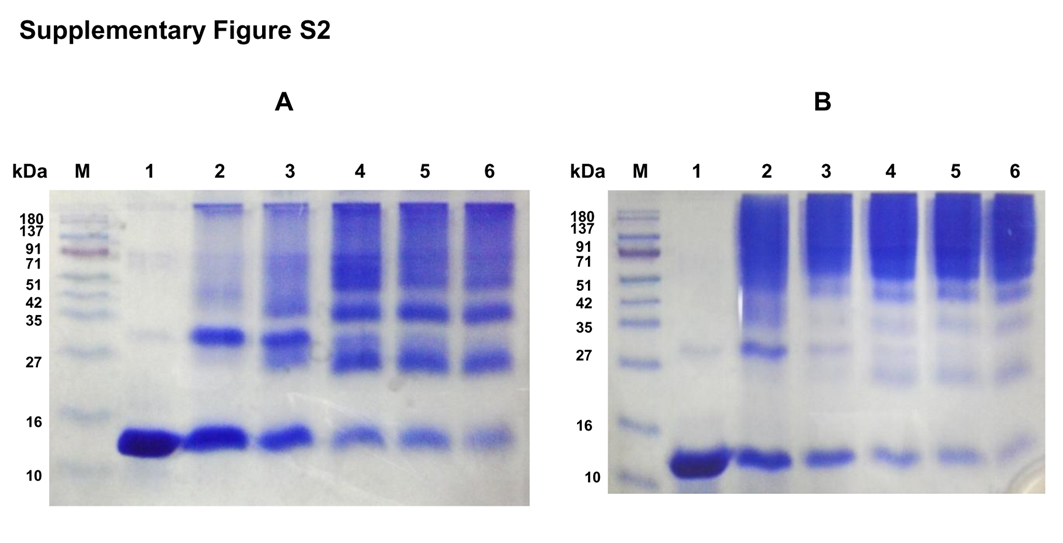

Supplement: Figure S2 — SDS-PAGE of glutaraldehyde treated α-LA (A) and BAMLET (B). α-LA and BAMLET were subjected to glutaraldehyde treatment as described under methods. Lane M: protein molecular weight marker; Lane 1: uncrosslinked α-LA/BAMLET; Lane 2, 3, 4, 5 and 6 contain the preparations crosslinked for 1, 5, 30, 60 and 360 min, respectively. (TIF) [file pone.0068390.s002.tif]
